# The metabolic side effects of 12 antipsychotic drugs used for the treatment of schizophrenia on glucose: a network meta-analysis

**DOI:** 10.1186/s12888-017-1539-0

**Published:** 2017-11-21

**Authors:** Yangyu Zhang, Yingyu Liu, Yingying Su, Yueyue You, Yue Ma, Guang Yang, Yan Song, Xinyu Liu, Mohan Wang, Lili Zhang, Changgui Kou

**Affiliations:** Department of Epidemiology and Biostatistics, School of Public Health, Jilin University, Changchun, Jilin Province 130021 China

**Keywords:** Antipsychotic drug, Glucose change, RCTs, Network meta-analysis

## Abstract

**Background:**

Antipsychotics have serious metabolic side effects on blood glucose. However, the comparative influence of these drugs on blood glucose levels has not been comprehensively evaluated. We conducted a network meta-analysis to create a hierarchy of the side effects of 12 antipsychotic drugs on changes in blood glucose levels.

**Methods:**

A systematic search of the PubMed, EMBASE and Cochrane databases (last search June 2016) was conducted to identify studies that reported randomized controlled trials (RCTs) comparing changes in blood glucose levels between patients receiving one of 12 antipsychotic drugs or a placebo for the treatment of schizophrenia or related disorders. The studies we searched were limited to those published in English. Two reviewers independently extracted data. The primary outcome of interest was changes in fasting glucose levels.

**Results:**

We included 47 studies with 114 relevant arms. Of the antipsychotic drugs, only olanzapine was associated with significantly increased glucose levels compared to a placebo (mean difference (MD) = 3.95, 95% confidence interval (CI) = 0.14 to 7.76). Moreover, olanzapine was associated with a significantly greater change in the glucose levels than ziprasidone (MD = 5.51, 95% CI = 1.62 to 9.39), lurasidone (MD = 5.58, 95% CI = 0.53 to 10.64) or risperidone (MD = 3.05, 95% CI = 0.87 to 5.22). Ziprasidone and lurasidone were associated with minimal glucose changes compared to the other antipsychotics.

**Conclusions:**

Olanzapine was associated with a significantly greater change in blood glucose levels than ziprasidone, lurasidone, risperidone or placebo treatment. The application of a hierarchy of glucose metabolism-related side effects may help clinicians tailor the choice of antipsychotic drug to meet the needs of individual patients.

**Electronic supplementary material:**

The online version of this article (10.1186/s12888-017-1539-0) contains supplementary material, which is available to authorized users.

## Background

Schizophrenia was ranked among the top 25 leading causes of disability worldwide in 2013 [1]. People with schizophrenia have mortality rates that are 2–3 times higher than the general population [2–4], which corresponds to a mortality gap of 10 to 20 years [5]. At least 60% of this premature mortality is due to cardiovascular diseases [6]. Diabetes mellitus, which is a significant risk factor of cardiovascular diseases [7], is a group of metabolic diseases in which a person has high blood glucose [8] that is characterized by chronic hyperglycemia [9] either because the body does not produce sufficient insulin or because the cells do not respond to the insulin that is produced. Type 2 diabetes mellitus (T2DM) comprises approximately 90% of diabetes cases, with the other 10% of cases due primarily to type 1 diabetes mellitus and gestational diabetes. The T2DM rates are estimated to be 2 to 3 times higher in people with schizophrenia than in the general population, with a prevalence of 10% to 15% [10, 11]. Several studies have indicated a progressive relationship between hyperglycemia and the cardiovascular event risk beginning with glucose levels well below the diabetic thresholds [12–14].

Second-generation antipsychotics (SGAs) have been extensively recommended as first-line agents for the treatment of schizophrenia. However, several studies have reported the presence of alterations in glucose metabolism during treatment with SAGs, particularly clozapine and olanzapine [15–19]. Additionally, an increasing number of studies have reported an increased risk of hyperglycemia, diabetes, lipid dysregulation and ketoacidosis in patients treated with SGAs [20–22]. These serious effects have gradually raised concerns about a possible association between these metabolic effects and treatment with these drugs [23].

Large epidemiological studies have provided conflicting evidence regarding the relative risk of diabetes observed in association with the use of different antipsychotics [24–28]. Few prior meta-analyses have assessed glucose changes in association with several types of antipsychotic drugs [29–31] since such comparisons have been limited by the inadequacy of direct head-to-head comparisons of specific drugs.

Compared with traditional meta-analyses, network meta-analyses provide a statistical framework that allows the incorporation of evidence from both direct and indirect comparisons from a network of studies of different drugs to evaluate their treatment effects. Direct and indirect comparisons of the efficacies of the 12 antipsychotic drugs and the glucose intolerance of some of these drugs have been performed [32, 33]. However, a comparison of the effects of these 12 antipsychotics on changes in glucose using a network meta-analysis with relatively sufficient evidence has not been performed to date. Hence, we performed a network meta-analysis to evaluate the comparative effects of 12 antipsychotic drugs on changes in the glucose levels with the objective of providing a point of reference for treatment selection.

## Methods

### Search strategy and selection criteria

Two independent reviewers searched the PubMed, EMBASE and Cochrane Central Register of Controlled Trials databases to identify studies published from January 1995 to June 2016 using medical subject headings (MeSH) and free words. The MeSH terms included “schizophrenia, glucose, randomized controlled trials (RCTs), amisulpride, aripiprazole, asenapine, sertindole, clozapine, haloperidol, ziprasidone, lurasidone, olanzapine, paliperidone, quetiapine and risperidone.” The search strategy was based on combining the MeSH terms for schizophrenia, glucose, the 12 types of oral antipsychotic drugs and RCTs with their free text variants (see Additional file 1 for search strategy details). Additionally, the reference lists of all eligible articles and recent systematic reviews were reviewed. We restricted the studies to those written in English.

Trials were considered eligible for inclusion if they (i) included people with schizophrenia or related disorders (defined by any diagnostic criteria) and had a duration of treatment that was no more than 1 year, (ii) compared any antipsychotic drugs with either placebo treatment or one of the other 12 antipsychotic drug monotherapies (amisulpride, aripiprazole, asenapine, sertindole, clozapine, haloperidol, ziprasidone, lurasidone, olanzapine, paliperidone, quetiapine and risperidone), (iii) reported fasting glucose as an outcome, and (iv) were RCTs. Trials were excluded if they (i) included schizophrenic patients with a concomitant medical illness, treatment resistance, or stable illness, (ii) were conducted in special populations, including patients with a mean age ≤ 14 years, obesity or metabolic diseases, (iii) evaluated an antipsychotic treatment outside of the recommended dosage range, and (iv) were conference papers.

### Data extraction

Two independent investigators (Zhang and Liu) reviewed the titles, abstracts and full articles that satisfied the inclusion criteria and independently extracted data into a predetermined database. The following information was extracted from each trial: first author’s name, year of publication, study design, numbers and ages of participants, diagnosis method, treatment regimen details, and changes in fasting glucose. Disagreements were discussed between investigators until a consensus was reached. When disagreements regarding article inclusion could not be resolved, third and fourth reviewers were consulted. We assessed the risk of bias using the Cochrane risk of bias tool with a low, high, and unclear risk of bias. The risk of bias was evaluated according to the following items: sequence generation, allocation concealment, blinding method, incomplete outcome data, selective reporting and other bias.

### Statistical analysis

We performed a network meta-analysis to analyze direct and indirect comparisons of the 12 different drugs and placebo treatment using a multivariate meta-analysis model with the STATA 13 statistical software (StataCorp. College Station, Texas, USA). A recent update in the multivariate meta-analysis procedure in STATA makes performing a network meta-analysis possible within a software that is frequently used for meta-analyses [34]. Forest plots were constructed using the R 3.2.3 software.

The outcome of interest was changes in glucose, which were defined as changes from baseline to endpoint. The mean glucose changes were used to compare the metabolic side effects of the 12 antipsychotic drugs and a placebo on glucose. Since these changes were continuous outcomes, the effect sizes were calculated as the mean differences (MDs) and 95% confidence intervals (CIs). The difference between the drugs was considered significant when the 95%CI for MD did not include 0 (equivalent to *P* < 0.05). To rank the effects of the treatment regimens, we used surface under the cumulative ranking (SUCRA) probabilities [35]. The SUCRA results were expressed as percentages to compare each intervention to an imaginary intervention, which was always the best intervention without uncertainty. A SUCRA of x% indicates that the intervention achieves x% of the effectiveness of the imaginary intervention; thus, larger SUCRAs indicate more preferable interventions [32]. We conducted inconsistency analyses to explore differences between the direct and various indirect effect estimates for the same comparison [36]. Inconsistency between direct and indirect comparisons may indicate transitivity that is not immediately obvious [37, 38]. Moreover, we calculated the differences between the direct and indirect estimates in all closed loops, with the simplest loop considered triangular (formed by three treatments compared with one another). The inconsistency factor (IF) was used to evaluate differences between the direct and indirect estimates for each of the comparisons in the loop. Additionally, we obtained a 95% CI and z-value for each IF [39, 40]. Notably, the IF is the logarithm of the ratio of two odds ratios (RoR) from the direct and indirect evidence in the loop, and RoR values approaching 1 indicate consistency between the two sources. Moreover, we conducted a subgroup analysis to explore the effects of duration in the short-term (≤12 weeks) and long-term groups (>12 weeks). Furthermore, a sensitivity analysis was performed by removing studies with less than 30 participants and placebo-controlled trials. Publication bias was assessed using a comparison-adjusted funnel plot.

## Results

### Search results

**Fig. 1:**
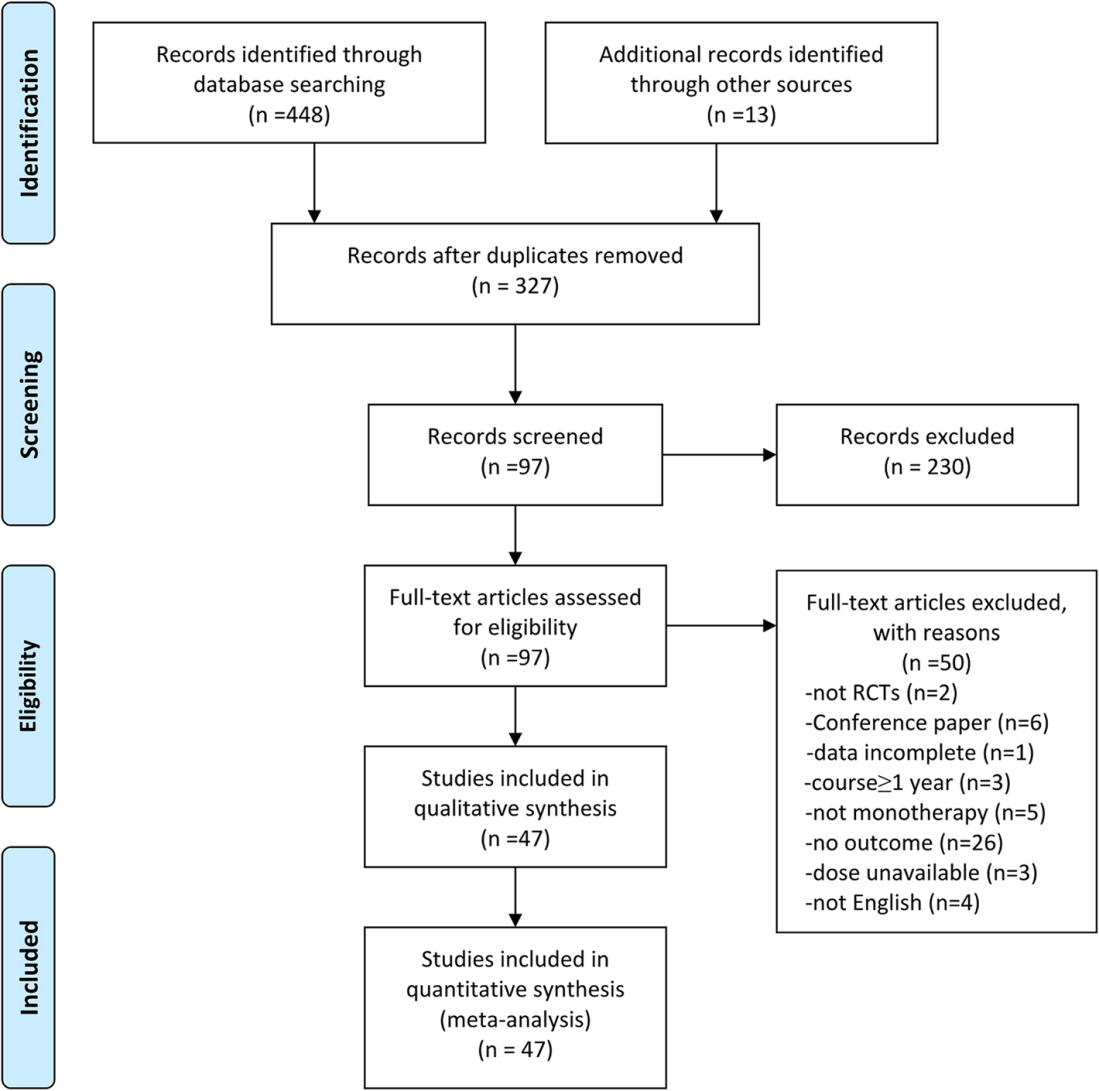
Flowchart diagram of randomized controlled trials reporting glucose change

The search strategy identified 448 studies. After a thorough review of the reference lists of all eligible articles and recent systematic reviews, we identified 13 additional studies. After excluding duplicate studies, the titles and abstracts of 327 studies were assessed; then, 97 articles were retrieved for more detailed review. Of these articles, only 47 reported the outcome of fasting glucose and had data available. The details of these 47 articles are shown in Additional file 2. Ten studies (10/47, 21.3%) provided details about randomization methods, and 26 studies (55.3% of all studies) were double-blind. Most studies did not describe the concealment details (see Additional file 3 for the risk of bias assessment). Within the included studies, the following treatment conditions were evaluated: placebo (10 studies), amisulpride (2 studies), aripiprazole (7 studies), asenapine (1 study), sertindole (2 studies), clozapine (6 studies), haloperidol (5 studies), ziprasidone (7 studies), lurasidone (4 studies), olanzapine (34 studies), paliperidone (7 studies), quetiapine (8 studies) and risperidone (21 studies). Thirty-two studies were two-arm trials. Eleven studies were three-arm trials. Three studies were four-arm trials, and one study was a five-arm trial. A total of 9846 individuals (aged from 15 to 65 years) were included in this meta-analysis. The flowchart of the literature retrieval process is shown in Fig. 1.

### Glucose changes

**Fig. 2:**
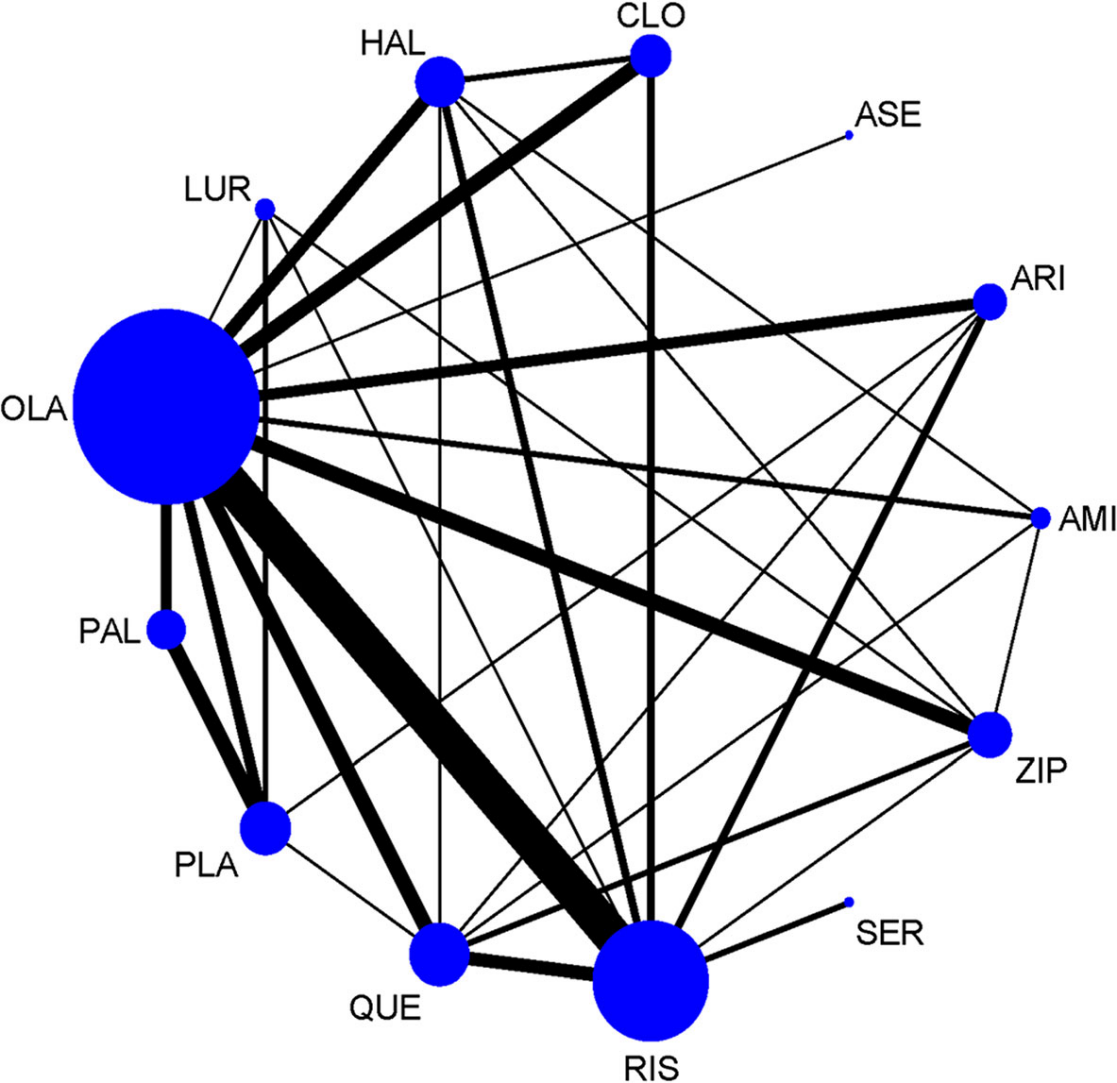
Network of treatment comparisons for glucose changes. PLA = placebo. AMI = amisulpride. ARI = aripiprazole. ASE = asenapine. CLO = clozapine. HAL = haloperidol. LURA = lurasidone. OLA = olanzapine. PAL = paliperidone. QUE = quetiapine. RIS = risperidone. SER = sertindole. ZIP = ziprasidone

The network of direct treatment comparisons for changes in fasting glucose is shown in Fig. 2. The sizes of the node reflect the number of corresponding trials. The lines link the direct comparisons, and the thickness of the lines represents the number of trials comparing the two therapies. The network plot indicated that olanzapine was included in the largest number of comparisons, followed by risperidone. Comparisons containing asenapine and sertindole were rare, whereas comparisons containing olanzapine and risperidone were frequently identified.

**Fig. 3:**
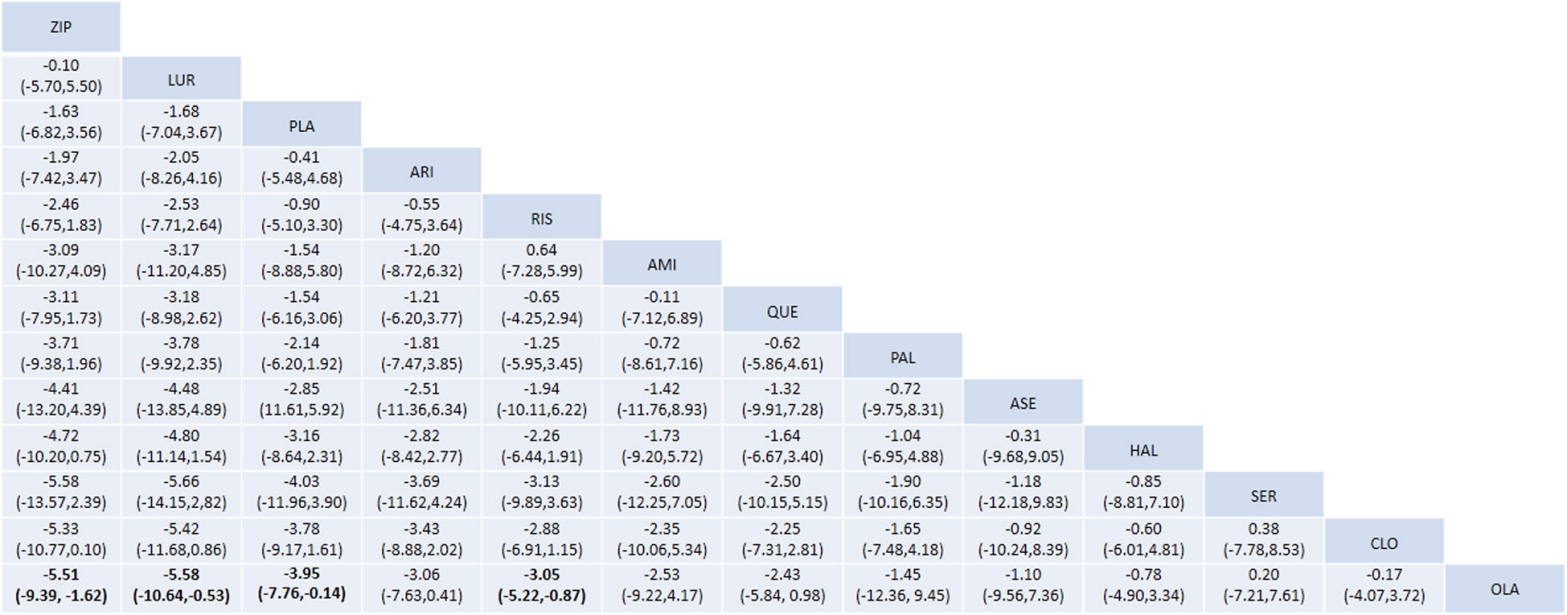
Glucose changes associated with antipsychotic drugs. Drugs are reported in order of their glucose change rankings. Comparisons between treatments should be read from left to right. The estimate is reported for the cell type in common between the column-defining treatment and the row-defining treatment. Mean differences (MDs) lower than 0 favor the column-defining treatment. Significant results are in bold. ZIP = ziprasidone. LUR = lurasidone. PLA = placebo. ARI = aripiprazole. RIS = risperidone. AMI = amisulpride. QUE = quetiapine. PAL = paliperidone. ASE = asenapine. HAL = haloperidol. SER = sertindole. CLO = clozapine. OLA = olanzapine

**Fig. 4:**
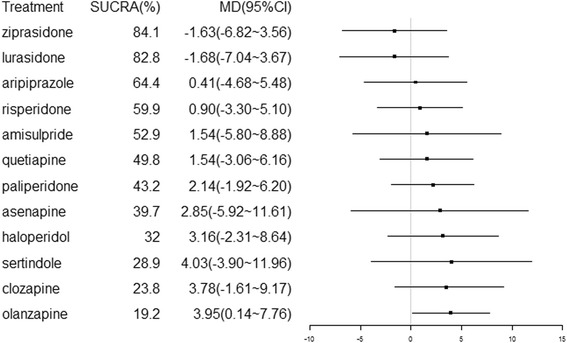
Forest plot for glucose changes associated with antipsychotics drugs compared with placebo treatment. Treatments are ranked based on their surface under the cumulative ranking (SUCRA) values

We created a hierarchy of effect sizes based on the SUCRA rankings for glucose change. Figure 3 shows the fasting glucose changes identified in association with the 12 antipsychotics. We used MDs to compare differences in the reported glucose changes. Regarding side effects related to blood glucose, only olanzapine was associated with a significant increase in glucose compared to placebo treatment (MD = 3.95, 95% CI = 0.14 to 7.76) (Fig. 4). Additionally, olanzapine was associated with a significantly greater change in glucose than ziprasidone (MD = 5.51, 95% CI = 1.62 to 9.39), lurasidone (MD = 5.58, 95% CI = 0.53 to 10.64) or risperidone (MD = 3.05, 95% CI = 0.87 to 5.22).

SUCRA values were used to determine the hierarchy of the antipsychotic treatments, which is shown in Fig. 4. A larger SUCRA value indicates a higher ranking for the drug. In our study, the SUCRA values indicated the following hierarchy among the 13 treatments: 69.4, 52.9, 64.4, 39.7, 28.9, 23.8, 32.0, 84.1, 82.8, 19.2, 43.2, 49.8 and 59.9% for placebo, amisulpride, aripiprazole, asenapine, sertindole, clozapine, haloperidol, ziprasidone, lurasidone, olanzapine, paliperidone, quetiapine and risperidone, respectively. These probabilities may inform the ranking of these treatments in terms of their glucose metabolism-related adverse effects.

### Inconsistency analysis of the obtained outcomes

To determine potential inconsistencies between the direct and indirect comparisons, we conducted an inconsistency analysis using glucose changes as the outcome of interest. The results indicated that there was no significant difference between the direct and indirect comparisons (χ^2^ = 37.49, *P* = 0.16). When we used node-splitting models, the direct and indirect comparisons between quetiapine and aripiprazole (*P* = 0.036) seemed to be inconsistent; however, no other inconsistencies were indicated. The results also showed that no significant inconsistencies were identified in any of the 31 loops, and all of the RoRs were compatible with zero inconsistency (RoR = 1). Nevertheless, several of the loops included values with high inconsistency (mean RoR larger than 2) that indicated that the direct estimates were twice as large as the indirect estimates or vice versa**.** Overall, no obvious inconsistencies were identified between the direct and indirect comparisons.

### Subgroup and sensitivity analyses

Through the subgroup analyses, we found some differences between short-term and long-term studies. In the short-term studies, no significant differences were identified between any antipsychotic drugs. However, in the long-term studies, comparisons between amisulpride, asenapine, sertindole, haloperidol, olanzapine, quetiapine, risperidone, and aripiprazole and between ziprasidone and olanzapine indicated significant differences.

The sensitivity analysis indicated stable results when studies with less than 30 participants were excluded; however, when we removed placebo-controlled trials, the rankings of the glucose metabolism-related effects changed. The comparison-adjusted funnel plot was symmetric around the zero line, indicating that there was no evidence of publication bias (see Additional file 4 for the funnel plot).

## Discussion

We attempted to integrate data from clinical trials as comprehensively as possible and performed an exhaustive comparison of the metabolic side effects of 12 antipsychotic drugs on blood glucose. Unlike traditional pairwise meta-analyses, network meta-analyses enable the performance of a simultaneous comparison of all included trials and can present a comprehensive and transparent picture of hierarchies among the 12 antipsychotic drugs. Network meta-analyses not only contain the outcomes of direct comparisons but also combine these results with those of indirect comparisons that are rarely reported in head-to-head RCTs, presenting an advantage relative to conventional meta-analyses. We present the first study to simultaneously compare side effects related to glucose changes that occur in association with 12 antipsychotic drugs in people with schizophrenia. Our study showed that of the drug treatments for schizophrenia, only olanzapine was associated with a significant increase in glucose-related side effects compared with placebo treatment. Olanzapine was also associated with a conspicuous and significant change in glucose compared with ziprasidone, lurasidone and risperidone. Based on the SUCRA probabilities, we created hierarchies of effect size for blood glucose metabolism. Ziprasidone and lurasidone were associated with fewer glucose-related side effects than any of the other antipsychotics and placebo treatment and were followed in ranking by aripiprazole, risperidone, amisulpride, quetiapine, paliperidone, asenapine and haloperidol. Olanzapine, clozapine and sertindole were associated with more substantial changes in the blood glucose levels.

This review strove to comprehensively and systematically review published evidence from a wide variety of sources. To minimize potential bias, two researchers independently searched and screened the literature, extracted available information and assessed the quality of the studies. The glucose concentrations evaluated in our network meta-analysis were entirely fasting glucose, and the evaluated doses of the drugs were within the specified ranges.

Hyperglycemia induced by antipsychotics was first reported in 1964 in association with phenothiazine derivatives [41]. A series of uncontrolled case reports revealed that hyperglycemia in schizophrenic patients was associated with antipsychotic treatment. After reviewing reports examining the association between hyperglycemia and diabetes and antipsychotic use, we found that associations with clozapine were identified most frequently, followed by olanzapine [25, 42–53]. A variety of published studies, including large retrospective database analyses, uncontrolled observational and controlled experimental studies, and RCTs, have reported associations between different types of atypical antipsychotics and changes in glucose levels. Although the patients treated with olanzapine and clozapine in these studies were more likely to develop diabetes, our results showed that only olanzapine was significantly associated with changes in glucose compared with placebo treatment, indicating a difference from the findings of other studies. This difference could have occurred because five of the six clozapine studies evaluated the use of short-term therapy (<12 weeks), whereas glucose levels might be more affected by long-term therapy [54]. In our study, the finding that sertindole was associated with elevated mean glucose levels was surprising; however, previous reports have also indicated small increases in glucose associated with sertindole [55] and an acceptable metabolic profile for this drug [56].

Lindenmayer et al. [57] conducted a study using modified glucose tolerance tests that showed that patients in the olanzapine-treated and clozapine-treated groups consistently exhibited significant increases in glucose levels relative to haloperidol-treated patients and untreated subjects in the control group. Reynolds et al. [58] hypothesized that SGAs might be responsible for incident diabetes cases, probably due to specific diabetogenic actions, particularly in association with drugs such as clozapine and olanzapine. However, although the first-generation antipsychotic haloperidol appeared to have less influence on glucose levels than olanzapine and clozapine in the current study, no obvious advantages were identified when haloperidol was compared to other SAGs.

Although a considerable number of studies have assessed the effect of risperidone treatment, few of these studies have identified side effects related to glucose, suggesting a limited risk of risperidone treatment-induced diabetes mellitus. Our study also found that risperidone had only a small effect on glucose levels. Similar to risperidone, few available studies suggested that quetiapine had an effect on glucose. Additionally, the lack of relevant published data from clinical trials made it difficult to conclude the risk of diabetes associated with the use of amisulpride. De Hert M et al. [30] found significant changes in glucose levels in association with short-term treatment (≤12 weeks) with asenapine and long-term (>12 weeks) treatment with paliperidone.

Lurasidone is a second-generation antipsychotic that was approved for use in the treatment of schizophrenia by the US Food and Drug Administration in October 2010. Recently, increasing numbers of clinical trials have published data related to lurasidone, and several of these studies have evaluated the impact of switching to lurasidone from other antipsychotics due to its adverse effects on metabolism. These studies suggested that treatment with lurasidone might be associated with a minimal risk of hyperglycemia and diabetes [59–62]. Although the amount of data from clinical trials is growing, the most recently published data have been obtained from large retrospective database analyses. At present, there is no evidence that treatment with ziprasidone and aripiprazole is associated with an increased risk of diabetes or other side effects related to glucose [23]. These results are consistent with our findings.

Our study has several limitations. First, drugs such as chlorpromazine and zotepine, which are beneficial for the treatment of schizophrenia, could have been included in our study; however, no data were available concerning their associations with the primary outcome of our study. Second, since asenapine, sertindole and amisulpride were rarely included in head-to-head trials, collecting a sufficient number of studies was difficult. Consequently, the results of comparisons including the 3 drugs had broader CIs and should be judged as less precise. Third, some randomized control trials evaluating the association between treatment for schizophrenia and glucose levels have reported inconsistent adverse effects or no side effects related to glucose metabolism. Fourth, the subgroup analyses of this study indicated that duration might be the source of heterogeneity; however, because the inclusion or exclusion criteria, study duration, dosage schedule, scales and assessment methods used may have differed across studies [63], the presence of heterogeneity cannot be fully ruled out. Fifth, although the duration of most studies included in this network meta-analysis was approximately 8 to 12 weeks, some studies were conducted for nearly 1 year, which was a relatively wide range of duration. Short-term and long-term trials may identify different effects on glucose metabolism. The subgroup analyses also indicated differences between the two groups. Sixth, our findings cannot be generalized to children and adolescents, stable patients or patients with predominant negative symptoms, all of whom were excluded to increase homogeneity.

## Conclusion

Our study presented a comprehensive and transparent picture of hierarchies among the 12 antipsychotics drugs on their effects of changes in blood glucose levels. Olanzapine was associated with a significantly greater change in glucose than ziprasidone, lurasidone, risperidone or placebo treatment. Therefore, cautious and individualized treatment decisions should be made by clinicians, and metabolism should be intensively monitored in schizophrenia patients when choosing antipsychotics for treatment to decrease the risks of developing hyperglycemia and diabetes.

## Additional files

Additional file 1: Search strategy. (DOCX 16 kb)

Additional file 2: Description of included studies. (DOCX 92 kb)

Additional file 3: Risk of bias assessment within studies. (DOCX 37 kb)

Additional file 4: Funnel plot. (DOCX 21 kb)

## Abbreviations

AMI: Amisulpride
ARI: Aripiprazole
ASE: Asenapine
CIs: Confidence intervals
CLO: Clozapine
DB: Double-blind
HAL: Haloperidol
IF: Inconsistency factor
LURA: Lurasidone
MDs: Mean differences
MeSH: Medical Subject Headings
OLA: Olanzapine
PAL: Paliperidone
PLA: Placebo
QUE: Quetiapine
RCTs: Randomized controlled trials
RIS: Risperidone
RoR: Odds ratios
SER: Sertindole
SGAs: Second-generation antipsychotics
SUCRA: Surface under the cumulative ranking
T2DM: Type 2 diabetes mellitus
ZIP: Ziprasidone
