# Supplementary material for: The metabolic side effects of 12 antipsychotic drugs used for the treatment of schizophrenia on glucose: a network meta-analysis
Source: BMC Psychiatry. 2017 Nov 21;17:373. doi: 10.1186/s12888-017-1539-0 (PMC5698995; doi:10.1186/s12888-017-1539-0)
Supplement: Supplementary file 1 — Search strategy. (DOCX 16 kb) [file 12888_2017_1539_MOESM1_ESM.docx]

Additional file 1: Search strategy term list

1 "Schizophrenia"[Mesh]

2 Schizophrenias[Title/Abstract])

3 Schizophrenic Disorders[Title/Abstract])

4 Disorder, Schizophrenic[Title/Abstract])

5 Disorders, Schizophrenic[Title/Abstract])

6 Schizophrenic Disorder[Title/Abstract])

7 Dementia Praecox[Title/Abstract])

8 #1 OR #2 OR #3 OR #4 OR #5 OR #6 OR #7

9 "Clozapine"[Mesh]

10 Clozaril

11 Leponex

12 amisulpride

13 olanzapine

14 Risperdal Consta

15 Consta, Risperdal

16 Risperidal

17 R-64,766

18 R 64,766

19 R64,766

20 R-64766

21 R 64766

22 R64766

23 "Risperidone"[Mesh]

24 #9 OR #10 OR #11 OR #12 OR #13 OR #14 OR #15 OR #16 OR #17 OR #18 OR #19 OR #20 OR #21 OR #22 OR #23

25  lurasidone

26"Haloperidol"[Mesh]

27 Haldol

28 quetiapine

29"Aripiprazole"[Mesh]

30 Abilify

31 OPC-14597

32 14597, OPC

33 OPC 14597

34 Aripiprazol

35 7-(4-(4-(2,3-dichlorophenyl)-1-piperazinyl)butyloxy)-3,4-dihydro-2(1H)-quinolinone

36 sertindole

37 ziprasidone

38 asenapine

39 Paliperidone

40 #25 OR #26 OR #27 OR #28 OR #29 OR #30 OR #31 OR #32 OR #33 OR #34 OR #35 OR #36 OR #37 OR #38 OR #39

41 #24 OR #40

42 Blood Sugar

43 Sugar, Blood

44 Glucose, Blood

45 Glucose

46 #42 OR #43 OR #44 OR #45

47 random*

48 Controlled Clinical Trial

49 "Randomized Controlled Trial" [Publication Type]

50 "Controlled Clinical Trial" [Publication Type]

51 #47 OR #48 OR #49 OR #50 OR #51

52 #8 AND #41 AND #46 AND #52
