# Supplementary material for: The metabolic side effects of 12 antipsychotic drugs used for the treatment of schizophrenia on glucose: a network meta-analysis
Source: BMC Psychiatry. 2017 Nov 21;17:373. doi: 10.1186/s12888-017-1539-0 (PMC5698995; doi:10.1186/s12888-017-1539-0)
Supplement: Supplementary file 2 — Description of included studies. (DOCX 92 kb) [file 12888_2017_1539_MOESM2_ESM.docx]

| **Additional file 2: Description of studies** | | | | | | | | |
| --- | --- | --- | --- | --- | --- | --- | --- | --- |
| Study | Reference | Design | Duration | N | Drug and dose | Patients | Diagnostic criteria | Age |
| [^1^](#_ENREF_1) | Kahn RS  2008 | Open-RCT | 1 year | 222 | haloperidol 1–4 mg, amisulpride 200–800 mg, olanzapine 5–20 mg, quetiapine 200–750 mg, or ziprasidone 40–160 mg. | schizophrenia, schizophreniform disorder or schizoaffective disorder(episode**)** | DSM-IV-TR | 18–40 |
| [^2^](#_ENREF_2) | Didier M 2010 | RCT(DB) | 6 weeks | 341 | quetiapine XR 600 mg/day; placebo | acute schizophrenia | DSM-IV | 18–65 |
| [^3^](#_ENREF_3) | Canuso CM 2010 | RCT(DB) | 6 week | 215 | paliperidone ER 6 mg/d; placebo | schizophrenia disorder | DSM-IV | 18-65 |
| [^4^](#_ENREF_4) | Bushe C 2010 | Open-RCT | six-month | 303 | olazapine 10–20 mg/day); quetiapine 300–700 mg/day | schizophrenia or schizoaffective disorder | DSM-IV | 41(9) |
| [^5^](#_ENREF_5) | Perez R 2009 | RCT(DB) | 1 year | 164 | haloperidol,10–20 mg/day; olanzapine 5–20 mg/day; risperidone 3–6 mg/day | psychotic disorder, schizophreniform disorder, schizophrenia, or schizoaffective disorder | DSM-IV | 27.0 (6.9) |
| [^6^](#_ENREF_6) | Krakowski M 2009 | RCT(DB) | 12 week | 87 | clozapine 3–9 mg/day; olanzapine 5–20 mg/day; risperidone 3–6mg/day . | schizophrenia or schizoaffective disorder | DSM-IV | 35.2(11.8) |
| [^7^](#_ENREF_7) | Ingole S 2009 | Open-RCT | 12 week | 60 | olanzapine 5mg/day; risperidone 3 mg/day | newly diagnosed | DSM-IV | 18–60 |
| [^8^](#_ENREF_8) | Hatta K 2009 | RCT(DB) | 8 week | 78 | risperidone 3mg/d;quetiapine 300mg/d; olanzapine 10mg/day;aripiprazole12 mg/day | acute-phase schizophrenia | ICD-10 | 18–64 |
| [^9^](#_ENREF_9) | Saddichha S 2008 | RCT(DB) | 6 week | 99 | olanzapine 16.5 ± 4.6 mg; haloperidol 13.4±3.6 mg;risperidone4.4±1.2 mg | first-episode schizophrenia | DSM-IV | 26.06±5.57 |
| [^10^](#_ENREF_10) | Meyer JM 2008 | RCT(DB) | 3 month | 226 | olanzapine 20.1mg/d; quetiapine 543.4mg/d; risperidone 3.9mg/d; ziprasidone 112.8mg/d | schizophrenia | n.i. | 18-65 |
| [^11^](#_ENREF_11) | Wu RR 2007 | Open-RCT | 8 week | 83 | clozapine (200–400mg/d);olanzapine (10–20mg/d); risperidone (2–5mg/d) | First-Episode Schizophrenia | DSM-IV | 18 to 45 |
| [^12^](#_ENREF_12) | McEvoy JP 2007 | RCT(DB) | 52-week | 267 | olanzapine 2.5–20 mg/day; quetiapine 100–800 mg/day; risperidone 0.5-4mg/day | Early Psychosis | DSM-IV | 16–40 |
| [^13^](#_ENREF_13) | Marder SR 2007 | RCT(DB) | 6 week | 285 | paliperidone ER 6 mg; placebo;olanzapine 10mg | acute episode of schizophrenia | DSM-IV | 41.6 |
| [^14^](#_ENREF_14) | Kane J 2007 | RCT(DB) | 6 week | 362 | paliperidone ER 9mg ; placebo; olanzapine 10mg once daily | acute episode | DSM-IV | 37.1±10.9 |
| [^15^](#_ENREF_15) | Davidson M 2007 | RCT(DB) | 6 week | 348 | paliperidone ER 9mg ; placebo; olanzapine 10mg | acute episode of schizophrenia, | DSM-IV | 37 |
| [^16^](#_ENREF_16) | Wu RR 2006 | RCT | 8 weeks | 83 | clozapine 200–400 mg/day;olanzapine10–20 mg/day; risperidone (2–5 mg/day) | first-episode schizophrenia | DSM-IV | 33 |
| [^17^](#_ENREF_17) | Vanelle JM 2006 | RCT(DB) | 8 weeks | 85 | amisulpride (200–600 mg/day);olanzapine (5–15 mg/day) | schizophrenia and presenting a depressive episode | DSM-IV | M:32.6±7.9；  F:36.5±8.0 |
| [^18^](#_ENREF_18) | Kinon BJ 2006 | RCT(DB) | 24 week | 207 | olanzapine10, 15, or 20 mg/d; ziprasidone | Schizophrenia or Schizoaffective Disorder | DSM-IV | 18-60 |
| [^19^](#_ENREF_19) | Chiu CC 2006 | Open-RCT | 2 week | 26 | olanzapine(10mg/d);risperidone2 mg/d | Atypical-naıve Schizophrenic | DSM-IV | 37.3 ± 8.3 |
| [^20^](#_ENREF_20) | Schoemaker J 2010 | RCT(DB) | 52 week | 1219 | asenapine5-10mg;olanzapine 10-20mg | schizophrenia or schizoaffective disorder | DSM-IV | M：36.8； F:36.2 |
| [^21^](#_ENREF_21) | Canuso CM 2010 | RCT(DB) | 6 week | 311 | paliperidone ER 3-12 mg/d; placebo | Schizoaffective Disorder | DSM-IV | 37.6 (9.2) |
| [^22^](#_ENREF_22) | Kumra S 2008 | Open-RCT | 12 week | 39 | Clozapine 403.1mg/d; olanzapine 26.2mg/d | schizophrenia, schizoaffective disorder | Kiddie-SADS | 15.6 |
| [^23^](#_ENREF_23) | Chan H 2007 | RCT(DB) | 4 week | 83 | Aripiprazole 15mg;Risperidone 6 mg | acute treatment of schizophrenia | DSM-IV | 35.2 |
| [^24^](#_ENREF_24) | Kane JM 2009 | RCT(DB) | 28 week | 566 | n.i. | schizophrenia | DSM-IV | 18-65 |
| [^25^](#_ENREF_25) | Newcomer JW 2009 | Open-RCT | 24 week | 393 | olanzapine 15.2mg/day; quetiapine 607.0mg/day; risperidone 5.2mg/day | schizophrenia | DSM-IV | 18-65 |
| [^26^](#_ENREF_26) | Wani RA 2015 | Open-RCT | 24 week | 47 | olanzapine 10–20mg/day; aripiprazole 5mg/day | schizophrenia | DSM-IV | M：29.84 (5.24);  F：29.71 (5.00) |
| [^27^](#_ENREF_27) | Bobo WV 2011 | Open-RCT | 6 month | 54 | olanzapine 5–20mg/day; Risperidone 2-6mg/day | schizophrenia, schizoaffective disorder or bipolar disorde | DSM-IV | 40.7(10.7) 40.4(11.1) |
| [^28^](#_ENREF_28) | Hert MD 2010 | Open-RCT | 24 week | 131 | Sertindole 12mg;Risperidone 4mg | schizophrenia | DSM-IV | 18-66 |
| [^29^](#_ENREF_29) | Smith RC 2009 | Open-RCT | 5 month | 46 | olanzapine 25.2mg/d; Risperidone 6.1mg/d | schizophrenia or pyschosis | DSM-IV | 41.22(7.2) ; 42.52(9.0) |
| [^30^](#_ENREF_30) | Hu S 2013 | Open-RCT | 12 week | 56 | paliperidone ER 7.55±2.27mg/day; olanzapine 15.87±3.34mg/day | schizophrenia | DSM-IV | 18–45 |
| [^31^](#_ENREF_31) | Grootens KP 2011 | RCT(DB) | 8-week | 61 | ziprasidone 80–160 mg/d; olanzapine 10–20 mg/d | schizophrenia, schizoaffective disorder, or schizophreniform disorder | DSM-IV | 18–40 |
| [^32^](#_ENREF_32) | Kelly DL 2003 | RCT | 8 weeks | 13 | clozapine 450 mg/day; olanzapine 50 mg/day | Treatment-Resistant Schizophrenia | DSM-IV | ni. |
| [^33^](#_ENREF_33) | Lindenmayer JP 2003 | RCT | 14-week | 73 | clozapine 500 mg/ day; olanzapine 20 mg/day; risperidone 8 mg/day; haloperidol 20 mg/day | schizophrenia or schizoaffective disorder | DSM-IV | n.i. |
| [^34^](#_ENREF_34) | Breier A 2005 | RCT(DB) | 28 weeks | 447 | ziprasidone 129.9±27.3 mg/day; olanzapine 11.3±2.8 mg/day | schizophrenia | DSM-IV | 18–75 |
| [^35^](#_ENREF_35) | Coppola D 2011 | RCT(DB) | 6-week | 116 | Paliperidone ER 1.5 mg; placebo | schizophrenia | DSM-IV | ≥18 |
| [^36^](#_ENREF_36) | Hardy TA 2011 | RCT | 12 weeks | 74 | olanzapine 5–20 mg; risperidone 4-10mg/d; | schizophrenia or schizoaffective disorder | n.i. | 18–65 |
| [^37^](#_ENREF_37) | De Hert M 2011 | Open-RCT | 12 weeks | 159 | sertindole 12 mg/d; risperidone 4mg/d | schizophrenia | n.i. | 18-65 |
| [^38^](#_ENREF_38) | Meltzer HY 2011 | RCT(DB) | 6 weeks | 354 | lurasidone 40 mg/d; olanzapine 15 mg/d | Schizophrenia, Schizoaffective Disorder and Schizophreniform Disorder | DSM-IV | 18–75 |
| [^39^](#_ENREF_39) | Potkin SG 2011 | RCT(DB) | 3-week | 270 | lurasidone 120mg once daily; ziprasidone 160 mg/day | chronic schizophrenia or schizoaffective disorder | DSM-IV | 18-70 |
| [^40^](#_ENREF_40) | Citrome L 2012 | RCT(DB) | 12 month | 525 | lurasidone 40 and 120mg/day; risperidone 2-6 mg/day | schizophrenia or schizoaffective disorder | DSM-IV | 18–75 |
| [^41^](#_ENREF_41) | Kaushal J 2010 | Open-RCT | 8 weeks | 60 | olanzapine 5 mg/d; risperidone 2 mg /d | schizophrenia, schizophreniform disorder or schizo-affective disorder | ICD-10 | 16–40 |
| [^42^](#_ENREF_42) | Jindal KC 2013 | RCT(DB) | 6 weeks | 60 | aripiprazole 12.5(2.55) mg; olanzapine 11.01(2.12) mg | schizophrenia | ICD-10 | 18-65 |
| [^43^](#_ENREF_43) | Ou JJ 2013 | RCT | 6-week | 255 | ziprasidone 138.2(28.6)mg/d; olanzapine19.0(2.3)mg/d | first-episode schizophrenia | DSM-IV | 18-45 |
| [^44^](#_ENREF_44) | Di FM 2014 | Open-label | 12-week, | 210 | quetiapine XR 400-800 mg/day); risperidone 4-6 mg/day | schizophrenia or schizoaffective disorder | DSM-IV | 18–65 years |
| [^45^](#_ENREF_45) | Kane JM 2014 | RCT(DB) | 12 weeks | 339 | aripiprazole once-monthly 400 mg; placebo | acute treatment of schizophrenia | DSM-IV-TR | 18-65 |
| [^46^](#_ENREF_46) | Robinson DG 2015 | RCT | 12 weeks | 198 | aripiprazole 5–30 mg/d; risperidone 1–6 mg/d | First-Episode Schizophrenia and Related Disorders | DSM-IV | 15–40 |
| [^47^](#_ENREF_47) | Nakamura M 2009 | RCT(DB) | 6 week | 144 | lurasidone 80mg/d; placebo | acute schizophrenia | DSM-IV | 30 - 50 |

**References**

**1.** Kahn RS, Fleischhacker WW, Boter H, et al. Effectiveness of antipsychotic drugs in first-episode schizophrenia and schizophreniform disorder: an open randomised clinical trial. *The Lancet.* 2008;371(9618):1085-1097.

**2.** Didier M, Karin H, Martin B. Safety and tolerability of once-daily extended release quetiapine fumarate in acute schizophrenia: pooled data from randomised, double-blind, placebo-controlled studies. *Human Psychopharmacology Clinical & Experimental.* 2010;25(2):103-115.

**3.** Canuso CM, Lindenmayer JP, Kosik GCI, Carothers J, Bossie CA, Schooler NR. A randomized, double-blind, placebo-controlled study of 2 dose ranges of paliperidone extended-release in the treatment of subjects with schizoaffective disorder. *J. Clin. Psychiatry.* 2010;71(5):587-598.

**4.** Bushe C, Sniadecki J, Bradley AJ, Poole Hoffmann V. Comparison of metabolic and prolactin variables from a six-month randomised trial of olanzapine and quetiapine in schizophrenia. *Journal of psychopharmacology (Oxford, England).* 2010;24(7):1001-1009.

**5.** Perez R. Glucose and lipid disturbances after 1 year of antipsychotic treatment in a drug-naïve population. *Schizophr. Res.* 2009;107(3):115-121.

**6.** Krakowski M, Czobor P, Citrome L. Weight gain, metabolic parameters, and the impact of race in aggressive inpatients randomized to double-blind clozapine, olanzapine or haloperidol. *Schizophr. Res.* 2009;110(1-3):95-102.

**7.** Ingole S, Belorkar NR, Waradkar P, Shrivastava M. Comparison of effects of olanzapine and risperidone on body mass index and blood sugar level in schizophrenic patients. *Indian J. Physiol. Pharmacol.* 2009;53(1):47-54.

**8.** Hatta K, Sato K, Hamakawa H, et al. Effectiveness of second-generation antipsychotics with acute-phase schizophrenia. *Schizophr. Res.* 2009;113(1):49-55.

**9.** Saddichha S, Manjunatha N, Ameen S, Akhtar S. Diabetes and schizophrenia – effect of disease or drug? Results from a randomized, double-blind, controlled prospective study in first-episode schizophrenia. *Acta Psychiatr. Scand.* 2008;117(5):342-347.

**10.** Meyer JM, Davis VG, Goff DC, et al. Change in metabolic syndrome parameters with antipsychotic treatment in the CATIE Schizophrenia Trial: Prospective data from phase 1. *Schizophr. Res.* 2008;101(1-3):273-286.

**11.** Wu RR, Zhao JP, Zhai JG, Guo XF, Guo WB. Sex difference in effects of typical and atypical antipsychotics on glucose-insulin homeostasis and lipid metabolism in first-episode schizophrenia. *J. Clin. Psychopharmacol.* Aug 2007;27(4):374-379.

**12.** McEvoy JP, Lieberman JA, Perkins DO, et al. Efficacy and tolerability of olanzapine, quetiapine, and risperidone in the treatment of early psychosis: a randomized, double-blind 52-week comparison. *The American journal of psychiatry.* 2007;164(7):1050-1060.

**13.** Marder SR, Kramer M, Ford L, et al. Efficacy and Safety of Paliperidone Extended-Release Tablets: Results of a 6-Week, Randomized, Placebo-Controlled Study. *Biol. Psychiatry.* 2007;62(12):1363-1370.

**14.** Kane J, Canas F, Kramer M, et al. Treatment of schizophrenia with paliperidone extended-release tablets: a 6-week placebo-controlled trial. *Schizophrenia research.* 2007;90(1-3):147-161.

**15.** Davidson M, Emsley R, Kramer M, Ford L. Efficacy, safety and early response of paliperidone extended-release tablets (paliperidone ER): results of a 6-week, randomized, placebo-controlled study. *Schizophr. Res.* 2007;93(1-3):117-130.

**16.** Wu RR, Zhao JP, Liu ZN, et al. Effects of typical and atypical antipsychotics on glucose-insulin homeostasis and lipid metabolism in first-episode schizophrenia. *Psychopharmacology (Berl.).* Jul 2006;186(4):572-578.

**17.** Vanelle JM, Douki S. A double-blind randomised comparative trial of amisulpride versus olanzapine for 2 months in the treatment of subjects with schizophrenia and comorbid depression. *European psychiatry : the journal of the Association of European Psychiatrists.* 2006;21(8):523-530.

**18.** Kinon BJ, Lipkovich I, Edwards SB, Adams DH, Ascher Svanum H, Siris SG. A 24-week randomized study of olanzapine versus ziprasidone in the treatment of schizophrenia or schizoaffective disorder in patients with prominent depressive symptoms. *Journal of clinical psychopharmacology.* 2006;26(2):157-162.

**19.** Chiu CC, Chen KP, Liu HC, Lu ML. The early effect of olanzapine and risperidone on insulin secretion in atypical-naive schizophrenic patients. *J. Clin. Psychopharmacol.* 2006;26(5):504-507.

**20.** Schoemaker J, Naber D, Vrijland P, Panagides J, Emsley R. Long-term assessment of Asenapine vs. Olanzapine in patients with schizophrenia or schizoaffective disorder. *Pharmacopsychiatry.* 2010;44(43):138-146.

**21.** Canuso CM, Schooler N, Carothers J, et al. Paliperidone extended-release in schizoaffective disorder: a randomized, controlled study comparing a flexible dose with placebo in patients treated with and without antidepressants and/or mood stabilizers. *J. Clin. Psychopharmacol.* 2010;30(5):487-495.

**22.** Kumra S, Kranzler H, Gerbino Rosen G, et al. Clozapine versus "high-dose" olanzapine in refractory early-onset schizophrenia: an open-label extension study. *Journal of child and adolescent psychopharmacology.* 2008;18(4):307-316.

**23.** Chan HY, Lin WW, Lin SK, et al. Efficacy and safety of aripiprazole in the acute treatment of schizophrenia in Chinese patients with risperidone as an active control: a randomized trial. *J. Clin. Psychiatry.* 2007;68(1):29-36.

**24.** Kane JM, Osuntokun O, Kryzhanovskaya L, et al. A 28-WEEK, RANDOMIZED, DOUBLE-BLIND STUDY OF OLANZAPINE VERSUS ARIPIPRAZOLE IN THE TREATMENT OF SCHIZOPHRENIA. *J. Clin. Psychiatry.* 2009;70(4):572-581.

**25.** Newcomer JW, Ratner RE, Eriksson JW, et al. A 24-week, multicenter, open-label, randomized study to compare changes in glucose metabolism in patients with schizophrenia receiving treatment with olanzapine, quetiapine, or risperidone. *J. Clin. Psychiatry.* 2009;70(4):487-499.

**26.** Wani RA, Dar MA, Chandel RK, et al. Effects of switching from olanzapine to aripiprazole on the metabolic profiles of patients with schizophrenia and metabolic syndrome: a double-blind, randomized, open-label study. *Neuropsychiatr. Dis. Treat.* 2015;11(default):685-693.

**27.** Bobo WV, Bonaccorso S, Jayathilake K, Meltzer HY. Prediction of long-term metabolic effects of olanzapine and risperidone treatment from baseline body mass index in schizophrenia and bipolar disorder. *Psychiatry Res.* 2011;189(2):200-207.

**28.** Hert MD, Mittoux A, He Y, Peuskens J. METABOLIC PARAMETERS IN A SUBSET OF PATIENTS IN THE SCOP STUDY. *Schizophr. Res.* 2010;117(2-3):500-501.

**29.** Smith RC, Lindenmayer JP, Davis JM, et al. Effects of olanzapine and risperidone on glucose metabolism and insulin sensitivity in chronic schizophrenic patients with long-term antipsychotic treatment: a randomized 5-month study. *J. Clin. Psychiatry.* 2009;70(11):1501-1513.

**30.** Hu S, Yao M, Peterson BS, et al. A randomized, 12-week study of the effects of extended-release paliperidone (paliperidone ER) and olanzapine on metabolic profile, weight, insulin resistance, and β-cell function in schizophrenic patients. *Psychopharmacology (Berl.).* 2013;230(1):3-13.

**31.** Grootens KP, van Veelen NM, Peuskens J, et al. Ziprasidone vs olanzapine in recent-onset schizophrenia and schizoaffective disorder: results of an 8-week double-blind randomized controlled trial. *Schizophrenia bulletin.* Mar 2011;37(2):352-361.

**32.** Kelly DL, Conley RR, Richardson CM, Tamminga CA, Carpenter Jr WT. Adverse Effects and Laboratory Parameters of High-Dose Olanzapine vs. Clozapine in Treatment-Resistant Schizophrenia. *Annals of Clinical Psychiatry.* 2003;15(3-4):181-186.

**33.** Lindenmayer JP, Czobor P, Volavka J, et al. Changes in glucose and cholesterol levels in patients with schizophrenia treated with typical or atypical antipsychotics. *Am. J. Psychiatry.* 2003;160(2):290-296.

**34.** Breier A, Berg PH, Thakore JH, et al. Olanzapine versus ziprasidone: results of a 28-week double-blind study in patients with schizophrenia. *Am. J. Psychiatry.* 2005;162(10):1879-1887.

**35.** Coppola D, Melkote R, Lannie C, et al. Efficacy and safety of paliperidone extended release 1.5 mg/day-A Double-Blind, placebo- and active-Controlled, study in the treatment of patients with schizophrenia. 2011.

**36.** Hardy TA, Henry RR, Forrester TD, et al. Impact of olanzapine or risperidone treatment on insulin sensitivity in schizophrenia or schizoaffective disorder. *Diabetes Obesity & Metabolism.* 2011;13(8):726–735.

**37.** De Hert M, Mittoux A, He Y, Peuskens J. Metabolic parameters in the short- and long-term treatment of schizophrenia with sertindole or risperidone. *European archives of psychiatry and clinical neuroscience.* Jun 2011;261(4):231-239.

**38.** Meltzer HY, Cucchiaro J, Silva R, et al. Lurasidone in the Treatment of Schizophrenia: A Randomized, Double-Blind, Placebo- and Olanzapine-Controlled Study. *Am. J. Psychiatry.* 2011;168(9):957-967.

**39.** Potkin SG, Ogasa M, Cucchiaro J, Loebel A. Double-blind comparison of the safety and efficacy of lurasidone and ziprasidone in clinically stable outpatients with schizophrenia or schizoaffective disorder ☆. *Schizophr. Res.* 2011;132(2–3):101-107.

**40.** Citrome L, Cucchiaro J, Sarma K, et al. Long-term safety and tolerability of lurasidone in schizophrenia: a 12-month, double-blind, active-controlled study. *Int. Clin. Psychopharmacol.* 2012;27(3):165-176.

**41.** Kaushal J, Bhutani G, Gupta R. Comparison of fasting blood sugar and serum lipid profile changes after treatment with atypical antipsychotics olanzapine and risperidone. *Singapore Med. J.* 2012;53(7):488-492.

**42.** Jindal KC, Singh GP, Munjal V. Aripiprazole versus olanzapine in the treatment of schizophrenia: a clinical study from India. *Int. J. Psychiatry Clin. Pract.* 2013;17(1):21-29.

**43.** Ou JJ, Xu Y, Chen HH, et al. Comparison of metabolic effects of ziprasidone versus olanzapine treatment in patients with first-episode schizophrenia. *Psychopharmacology.* 2013;225(3):627-635.

**44.** Di FM, Montagnani G, Trespi G, Kasper S. Extended-release quetiapine fumarate (quetiapine XR) versus risperidone in the treatment of depressive symptoms in patients with schizoaffective disorder or schizophrenia: a randomized, open-label, parallel-group, flexible-dose study. *Int. Clin. Psychopharmacol.* 2014;29(3):166-176.

**45.** Kane JM, Peters Strickland T, Baker RA, et al. Aripiprazole once-monthly in the acute treatment of schizophrenia: findings from a 12-week, randomized, double-blind, placebo-controlled study. *The Journal of clinical psychiatry.* 2014;75(11):1254-1260.

**46.** Robinson DG, Gallego JA, John M, et al. A Randomized Comparison of Aripiprazole and Risperidone for the Acute Treatment of First-Episode Schizophrenia and Related Disorders: 3-Month Outcomes. *Schizophr. Bull.* 2015;41(6).

**47.** Nakamura M, Ogasa M, Guarino J, et al. Lurasidone in the treatment of acute schizophrenia: A double-blind, placebo-controlled trial. *Journal of Clinical Psychiatry.* 2009;70(6):829-836.
