# Supplementary figures and images for: The metabolic side effects of 12 antipsychotic drugs used for the treatment of schizophrenia on glucose: a network meta-analysis
Source: BMC Psychiatry. 2017 Nov 21;17:373. doi: 10.1186/s12888-017-1539-0 (PMC5698995; doi:10.1186/s12888-017-1539-0)

**Additional file 3: Risk of bias summary: judgements about each bias item for each study**


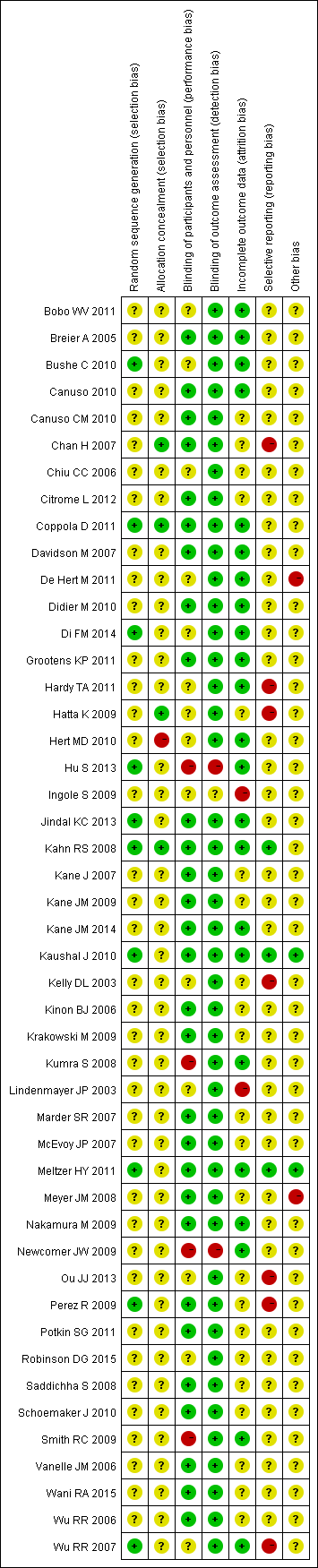

Supplement: Supplementary file 3 — Risk of bias assessment within studies. (DOCX 37 kb) [file 12888_2017_1539_MOESM3_ESM.docx]
