# Supplementary material for: The metabolic side effects of 12 antipsychotic drugs used for the treatment of schizophrenia on glucose: a network meta-analysis
Source: BMC Psychiatry. 2017 Nov 21;17:373. doi: 10.1186/s12888-017-1539-0 (PMC5698995; doi:10.1186/s12888-017-1539-0)
Supplement: Supplementary file 4 — Funnel plot. (DOCX 21 kb) [file 12888_2017_1539_MOESM4_ESM.docx]

**Additional file 4: Comparison-adjusted funnel plot**

OLA=olanzapine, PAL=paliperidone, QUE=quetiapine, RIS=risperidone, ZIP=ziprasidone, SER=sertindole, PLA= placebo, ARI=aripiprazole, AMI=amisulpride. LURA=lurasidone.
